# A Cascara-Infused Caffeine Drink as a Social Beverage

**DOI:** 10.3390/molecules30132749

**Published:** 2025-06-26

**Authors:** Magdalena Słowik-Borowiec, Bernadetta Oklejewicz, Maciej Wnuk

**Affiliations:** Faculty of Biotechnology, Collegium Medicum, University of Rzeszow, 1 Pigonia St., 35-310 Rzeszów, Poland; mslowik@ur.edu.pl (M.S.-B.); boklejewicz@ur.edu.pl (B.O.)

**Keywords:** coffee, cascara tea, social drink, specialty coffee

## Abstract

Specialty coffee commercialization has experienced a consistent upward trend over the past several years. The prevalence of specialty coffee consumption has increased considerably, particularly among younger demographics and people who engage in physical activities. Sellers are actively involved in the development of innovative formulas and modifications to maintain the competitiveness of their product in the market. Here, we propose a naturally infused caffeine drink with cascara extract as an alternative social beverage. This beverage was formulated using extracts derived from Arabica Ethiopia coffee beans and coffee cherry shells. The final cascara-infused caffeine drink comprises a 50% Ethiopian Arabica coffee infusion and a 50% coffee cherry shell infusion. This beverage is characterized by an average caffeine content of 0.28 mg/mL, a caffeic acid content of 0.24 mg/mL, and a chlorogenic acid content of 0.13 mg/mL. Furthermore, 100 mL of the cascara-infused coffee drink is enriched with polyphenol compounds at an amount of 80.6 mg of Gallic Acid Equivalents per liter (mg GAE/L), including 67.6 mg of catechin equivalent per liter (mg CAE/L) flavonoids. The formulation of the infused caffeine drink contains natural sugars such as glucose, sucrose, and fructose, in amounts of 0.17 mg/mL, 0.97 mg/mL, and 1.66 mg/mL, respectively. The developed procedure has the potential to enhance the coffee-sale market.

## 1. Introduction

Coffee is one of the most popular beverages in the world, having gained popularity in many cultures due to its ability to provide consumers with a quick source of energy, improve concentration, and reduce exhaustion [1,2]. Caffeinated beverages also have an important social role [2]. Many people are looking for new taste experiences, including growing expectations for new taste sensations [3]. For this reason, the beverage industry is intensively exploring new innovative methods for the creation of coffee-based beverages [4]. Increased consumer awareness and nutrition education also contribute to this trend. Many consumers are looking for new taste experiences and pay close attention to how coffee is prepared, the type of beans used, and the brewing method [5]. Growing nutritional awareness has contributed to the increase in cafés offering specialty coffee. The main characteristics of “specialty coffee” consumption culture are attention to the quality of the beans; the method of cultivation and origin; the production method, taking into account the working conditions of farmers and environmental protection; the characteristic taste and aromatic profile; and innovations in brewing and serving [6]. The search for new sensory and taste experiences by café customers represents an attractive market for coffee producers. New solutions and coffee blends also present market opportunity for small craft coffee roasters and producers who grow coffee based on the principles of sustainable agriculture. Newly developed beverages can also be a natural alternative to popular energy drinks that contain, in addition to caffeine, ingredients such as taurine, inositol, simple sugars, B vitamins, flavorings, carbon dioxide, preservatives, and acidity regulators [7].

A promising alternative is a beverage augmented with caffeine sourced from cascara, which are dried coffee husks. Cascara, traditionally utilized in certain cultures for brewing purposes, is abundant in natural bioactive compounds such as caffeine, antioxidants, and vitamins [8]. Therefore, it is not only a source of energy but also has potential health benefits. Furthermore, the use of cascara is in line with sustainability trends because it is a by-product of the coffee industry that has often been wasted until now [8]. In the present short communication, we propose a cascara-infused caffeine drink as an alternative social drink.

## 2. Results and Discussion

The content of caffeine, caffeine acid, and chlorogenic acid compounds determined in cascara-infused caffeinated beverages was calculated as the average of two parallel determinations. The results were compared with a pure coffee extract and a pure sample (cascara) of the coffee cherry shell extract. The identification of the compounds was conducted by comparing the retention times of the analyzed peaks with those of the standards, as well as by comparing them to the spectra of the standards for caffeine, caffeic acid, and chlorogenic acid. For quantitative determinations, calibration standard solutions (with a purity of 98%, Sigma-Aldrich GmbH, Hamburg, Germany) at concentrations of 1, 5, 10, 50, and 100 µg/mL were prepared from the standard stock solution (200 µg/mL) by appropriate dilution processes using the mobile phase. The peak height obtained for each concentration of the tested compounds was plotted against the concentration, and then the regression equations were calculated. The following linear standard equations, y = 0.7045x + 0.0396 (R^2^ = 1.00), y = 1.2192x + 5.9209 (R^2^ = 0.99), and y = 0.2015x − 0.1522 (R^2^ = 0.99), were obtained for caffeine, caffeic acid, and chlorogenic acid, respectively. The LOD and LOQ values in this study were 0.4 μg/mL and 1.0 μg/mL, respectively.

The results are presented in Table 1. The analyses carried out showed that the extract obtained from 20 mg/mL of the coffee cherry shell contained, on average, 8 times less caffeine, 13 times less caffeine acid, and 2 times less chlorogenic acid compared to the extract obtained from 20 mg/mL of ground coffee beans. This result is consistent with previous observations that coffee cherry shells are low in caffeine [9,10].

The determination of the total polyphenol content was performed using the Folin-Ciocalteu method. A calibration curve was constructed using gallic acid in the concentration range 25–100 µg/mL. Calculations were performed based on the equation obtained for the standard curve: y = 7.6659x + 0.2262, where y—was the absorbance of the sample, and x—the concentration of gallic acid (R^2^ = 0.99). The total polyphenol content is expressed as the mg of gallic acid per 100 g of dry matter. The flavonoid content in the coffee beverage samples was calculated based on the calibration curve (equation: y = 1.5199x + 0.0376, R^2^ = 0.99) for catechin as a standard at various concentrations (0.5–0.0 mg/mL). The total flavonoid content was expressed as the mg of catechin equivalent to the CAE per 100 g of dry matter. Three repetitions were made from each extract. The analysis results are presented in Table 2.

In the case of polyphenol content analysis, it was shown that the coffee extract contained, on average, 9 times more polyphenols than the coffee cherry shell extract. A comparison of the flavonoid content revealed that the flavonoid content in the coffee cherry shell extract was more than 50 times lower than in the coffee extract.

At the same time, the pH measurement showed, in line with previous data, that the pH of the pure coffee was more acidic than that of the coffee cherry shell extract.

In the case of the cascara-infused caffeinated beverage, the caffeic acid content was equal to or lower, and the chlorogenic acid content was 1.5 times lower compared to the extract obtained from the ground coffee beans, but interestingly, the chlorogenic acid content was comparable in both drinks. The pH of the cascara-infused caffeinated beverage was also higher than that of pure coffee.

The comparative analysis of the sugar content of the test beverages also revealed interesting results. Sugar identification was carried out according to retention times. The individual quantification of sugars was performed by external standardization using analytical curves comprised of six concentrations (0.025, 0.1, 0.5, 1.0, 2.5, and 5.0 mg/mL) of a mixture with glucose, sucrose, and fructose (Chempur, Piekary Śląskie, Poland) obtained through the dilution of analytical standards in ultra-pure water. Equations of the calibration curves with linear coefficients for each sugar confirm the linearity of the method: y = −0.3647x^2^ + 5.7464x + 0.8031, R^2^ = 0.997 (sucrose), y = −0.1666x^2^ + 4.2581x + 0.9645, R^2^ = 0.994 (fructose), and y = −0.4116x^2^ + 5.9947x + 0.8186, R^2^ = 0.997 (glucose). The LOD of the proposed method was 0.010 mg/mL, and the LOQ was 0.025 mg/mL for the three sugar types analyzed. The sugar content in the coffee infusions was calculated as the average of two parallel determinations. The results are presented in Table 3. The cascara-infused caffeinated beverage contained more than 30 times more fructose and glucose compared to the pure coffee extract, while the saccharose level was comparable between the two beverages.

As shown above, developing recipes for coffee drinks infused with different extracts can reduce the caffeine content, lower acidity, enrich their taste and aroma, improve hydration, and even improve the sweetness of the drink. Such modifications for coffee drinks can be of particular importance in the diets of people suffering from ulcers and heart disease. As can be seen, coffee stimulates stomach acid secretion, which can lead to dyspepsia (poor digestion, discomfort, nausea, heartburns, eructation, and flatulence), esophageal burns, gastritis or ulcers, and gastroesophageal reflux disease [10]. On the other hand, a human diet enriched with the regular consumption of a small amount of coffee can also have a positive effect on the body. An investigation conducted with a substantial cohort of Italian adults demonstrated that moderate intake (3–4 cups per day) of Italian-style coffee (with each cup measuring 30 mL, corresponding to the size of an Italian espresso) was correlated with reduced risks of overall mortality and, more specifically, mortality due to cardiovascular disease [11]. It has also been shown that coffee reduces the risk of diabetes, depression, and certain neurodegenerative diseases, lowers fat levels, stimulates DNA repair processes, and has an effect on epigenetic processes [11,12,13,14,15]. It is generally accepted that caffeine stimulates diuresis, although there is no evidence of dehydration with moderate daily coffee intake [16].

In many countries, it is common to drink between one and even five cups a day [11,17,18]. Therefore, the development an infused beverages would, on the one hand, provide a safe daily dose of caffeine as a natural energy boost and, at the same time, ensure its regular consumption by consumers. Coffee cherry shell infusions showed different levels of caffeine, caffeic acid, chlorogenic acid, total polyphenols, and flavonoids compared to solutions created using coffee and its shell. In general, the content of polyphenols, including flavonoids, caffeine, and caffeic acid, depended on the content of coffee extract in the drink. In turn, the addition of the coffee cherry shell led to a reduction in the acidity of the drink and gave it a sweeter, milder taste. The cascara-infused caffeine drink was richer in polyphenolic compounds than traditional coffee cherry shell tea. Heeger et al. [10] demonstrated that the aqueous extraction of coffee pulps disclosed a total polyphenolic concentration ranging from 4.9 to 9.2 mg gallic acid equivalents (GAEs) per gram of dry matter (DM). The same researchers demonstrated that a Bourbon cultivar originating from Congo and the Maragogype cultivar exhibited the highest concentrations of caffeine, measuring 6.5 and 6.8 mg/g of dry matter (DM), respectively [9]. The caffeine drink infused with cascara will ensure the presence of polyphenols in the drink that are not found in coffee extracts [9,19]. Detailed LC-QTOF analyses of the polyphenol content in coffee pulp revealed the presence of 35 polyphenolic compounds [17,20]. These results have been observed and confirmed by several research groups [17,18]. As shown, one of the main compounds present in coffee cherry shells is hydroxycinnamic acids. Among them, the highest proportion is made up of caffeoylquinic acids, which constitute a large part of cascara and have been reported to constitute about 2.5% of the dry matter of coffee pulp [19].

Another positive effect of enriching the coffee with cascara extract was an increase in its natural sweetness. The main sugar fraction responsible for the sweetness of the drink is fructose, which is also a simple sugar with a low glycemic index, and its metabolism occurs without the involvement of insulin [21]. As shown by Jiamjariyatam, the sugar content of cascara infusions can be up to 56% per 250 mL of the sample [21]. Our results are also consistent with previous data indicating that the Cascaria fruit contains sugars such as fructose, glucose, and sucrose [17]. A study conducted by Pua et al. [17] on five cascara samples quantified by HPLC-DAD and HPLC-ELSD techniques showed that the fructose content ranged from 0.105 to 7.621 mg/mL, and the sucrose content ranged from 0.127 to 0.443 mg/mL [17]. They also observed that the glucose level was rather low compared to the other sugars, ranging from 0.007 to 1.630 mg/mL [17]. The authors explain that the sugar content may vary and depend on pulp processing. At the same time, the authors point to the possible contribution of spontaneous fermentation processes during the drying of wet pulp [17].

It is also important to note that coffee pulp extract has cytoprotective and antioxidant properties [22]. Cañas et al. assessed how the in vitro digestion of coffee pulp and extract affects the phenolic profile, radical scavenging, antioxidant activity, and cytoprotective properties in IEC-6 and HepG2 cells [22]. They showed that the coffee pulp and its extract contained a high amount of caffeine and phenolic compounds, mainly phenolic acids (3′,4′-dihydroxycinnamoyl and 3,4-dihydroxybenzoic acid) and flavonoids (3,3′,4′,5,7-pentahydroxyflavone derivatives). Simulated digestion resulted in an increased antioxidant capacity, and the extract coffee pulp showed free radical scavenging activity even after in vitro digestion [22]. Simultaneously, they did not observe any induction of cytotoxicity in intestinal and liver cells. Furthermore, research has demonstrated that the administration of coffee pulp mitigated a reduction in glutathione, thiol groups, and the enzymatic activities of superoxide dismutase and catalase induced by tert-butyl hydroperoxide in IEC-6 and HepG2 cells [22].

## 3. Materials and Methods

### 3.1. Materials

In the present study, we used Ethiopian Arabica beans (supplier: The Barn GmbH, Berlin, Germany) and Carmen Estate cascara containing Geisha, Caturra, and Catuai coffee cherry shells (Coffeelab, Warsaw, Poland).

### 3.2. The Procedure for the Preparation of the Cascara-Infused Caffeinated Beverage

**Figure 1:**
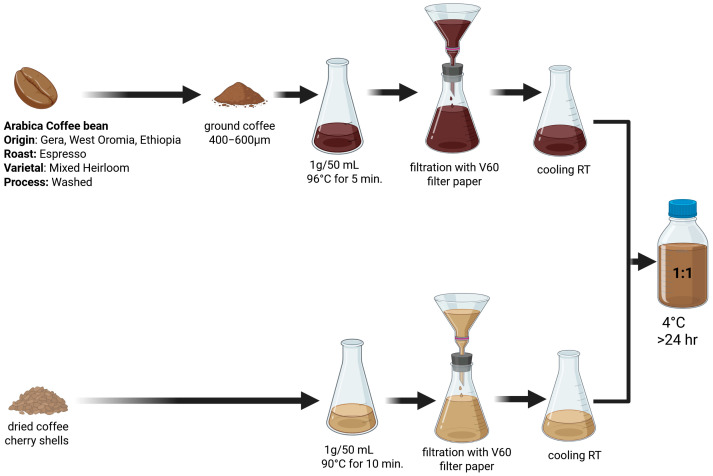
A diagram illustrating the procedure for preparing a cascara-infused caffeine drink (see details below).

The methodology for the preparation of a cascara-infused caffeinated beverage encompasses the following series of steps (Figure 1). Initially, a brew is prepared using Ethiopian Arabica coffee (origin: Gera, West Oromia; roast: espresso; varietal: mixed heirloom; process: washed) which is derived from ground beans with a particle size range of 400 µm to 600 µm. The ground coffee is utilized at a concentration of 1 g per 50 mL of filtered water and brewed at a temperature of 96 °C for a duration of 5 min, subsequently undergoing filtration through V60 filter paper, and then allowed to cool to ambient room temperature, specifically at 25 °C. Concurrently, an infusion of dried coffee cherry shells is prepared, with the shells being weighed at a rate of 1 g per 50 mL of filtered water and steeped at a temperature of 90 °C for a period of 10 min, followed by cooling to a temperature of 25 °C. Subsequently, the prepared coffee infusion is amalgamated with the cherry shell infusion in a 1:1 ratio. Finally, the resultant beverage is cooled to a temperature of 4 °C for a minimum duration of 24 h. Samples of the cascara-infused caffeine prepared according to the procedure described above were analyzed for caffeine (PubChem CID 2519), caffeic acid (PubChem CID 689043), chlorogenic acid (PubChem CID 1794427), and polyphenolic compounds including flavonoids and sugars such as glucose (PubChem CID 5793), sucrose (PubChem CID 5988), and fructose (PubChem CID 2723872).

### 3.3. High-Performance Liquid Chromatography Analysis

Caffeine, caffeic acid, and chlorogenic acid quantifications were conducted via reversed-phase high-performance liquid chromatography, utilizing UV-VIS/DAD detection (Dionex, model Ultimate 3000, Germering, Germany) in the isocratic elution mode. The mobile phase comprised (A) methanol (Honeywell Specialty Chemicals Seelze GmbH, Seelze, Germany) and (B) water in a 50:50 (*v*/*v*). The eluent’s flow rate was maintained at 0.5 mL/min, and the injection volume was set to 5 μL. Sample separation was conducted using an ACE 5 C18 column (Phillipsburg, NJ, USA) configured with 5 μm (250 mm × 4.6 mm) shell particles and a pre-column. The chromatographic column oven was operated at a consistent temperature of 25 °C. Spectrophotometric quantification of the target compounds was achieved at a wavelength of λ = 190–360 nm [23,24]. The Chromeleon 6.8 software (Dionex Corporation, Sunnyvale, CA, USA) facilitated data processing, and the total duration of the analysis was 10 min. The chromatogram of the analytical standards is depicted in Figure S1.

The determination of the sugar content (fructose, glucose, and sucrose) in the coffee infusions was performed on an HPLC Ultimate 3000 system (Dionex, Germering, Germany) consisting of a charged aerosol detector ultra RS (ESA, Chelmsford, MA, USA). Samples were separated on a Repromer H column packed with 9 µm shell particles (300 mm × 8 mm). Chromatographic analysis was performed at 25 °C with the mobile phase: for (A) acetonitrile (J.T. Baker, Malinckrodt Baker B.V., Deventer, The Netherlands) and (B) water (3:97, *v*/*v*) in the isocratic elution mode, the flow rate of the eluents was 0.50 mL/min, and the injection volume was 10 μL. The nitrogen (99.99%) gas flow rate was regulated automatically at 35 psi and monitored by the CAD device [25,26]. The data was processed with the Chromeleon 6.8 software (Dionex Corporation, USA). The total time of analysis was 20 min. An sample chromatogram is presented in Figure S2.

### 3.4. Colorimetric Analysis

The concentration of polyphenols within the analyzed samples was quantified utilizing the Folin–Ciocalteu reagent, which was diluted in a 1:10 ratio with H_2_O in conjunction with the addition of 7.5% Na_2_CO_3_ (Alchem, Wrocław, Poland). Subsequently, readings were recorded following a 15 min incubation period of the samples in a dark environment at 45 °C. The absorbance measurements were conducted at a wavelength of 765 nm (Tecan Infinite M200 PRO, Männedorf, Switzerland) [27].

The flavonoid concentration was assessed with aluminum chloride colorimetric assay following the addition of 5% NaNO_2_ (Alchem, Poland) to the extracts. The samples were subsequently mixed and maintained at ambient temperature, after which 10% AlCl_3_ (Alchem, Poland) was introduced. Following an additional 6 min incubation period, 100 µL of 1 M NaOH (Alchem, Poland) and 50 µL of ultra-pure H_2_O were supplemented to the samples, and absorbance measurements were conducted at a wavelength of 510 nm [28].

## 4. Conclusions

Our results suggest that enriching a coffee drink with cascara infusions can be a new alternative for consumers who are interested in the offerings of specialty coffee sellers. Our proposal is a response to the changing needs and preferences of consumers.

Although it is not a coffee drink in the classic sense, it still contains caffeine, so it has a stimulating effect, but in a milder way than traditional coffee. It also contains other phytonutrients that have a positive effect on the human body, including caffeic and chlorogenic acids, polysaccharides, and polyphenols, which remove excess free radicals, eliminating their harmful effect on the body and reducing the risk of lifestyle diseases. One cup (100 mL) of a beverage consisting of 50% Ethiopian Arabica coffee infusion and 50% coffee cherry husk infusion contains an average of 0.28 mg/mL, caffeic acid at 0.24 mg/mL and chlorogenic acid at 0.13 mg/mL, and is also enriched with polyphenolic compounds with an average content of 80.6 mg GAE/L, including flavonoids at 67.6 mg CAE/L and natural sugars such as glucose, sucrose, and fructose in the amount of 0.17 mg/mL, 0.97 mg/mL, and 1.66 mg/mL, respectively.

The proposed drink combines tradition with modernity and is a sustainable approach for the production and use of different parts of coffee beans, proposing products that not only quench thirst, but also support health and care for the environment, and offer a unique taste experience. However, we would like to emphasize that although our drink has many advantages over pure black tea and black coffee, the consumption of caffeinated beverages should always be rationalized according to the individual needs and physiological condition of the consumer.

## Figures and Tables

**Table 1:** Caffeine, caffeic acid, chlorogenic acid, polyphenols, and flavonoid content in sample drinks.

Beverage	Concentration (μg/mL)		
	Caffeine	Caffeic Acid	Chlorogenic Acid
Cascara	52.95 ± 1.23	29.79 ± 0.65	5. 59 ± 0.53
Ethiopian Arabica beans	449.97 ± 6.33	394.56 ± 29.42	13.26 ± 1.47
Cascara-infused caffeine drink	281.06 ± 16.47	247.76 ± 33.33	13.36 ± 2.81

The concentration values (caffeine, caffeic acid, or chlorogenic acid) within all samples are statistically significant *p* < 0.01 (ANOVA) with Tukey’s test, *n* > 3.

**Table 2:** pH value and total polyphenol and flavonoid content in coffee beverages.

Beverage	Polyphenols (µg of GAE/mL of Extract)	Flavonoid (µg of CAE/mL of Extract)	pH
Cascara	136.60 ± 3.5	23.14 ± 1.0	5.8 ± 0.3
Ethiopian Arabica beans	1256.43 ± 30.3	1238.29 ± 10.1	4.8 ± 0.2
Cascara-infused caffeine drink	806.49 ± 15.7	675.92 ± 6.28	5.4 ± 0.2

GAE—the total phenolic content expressed in terms of the gallic acid equivalent. CAE—the total flavonoid content expressed in terms of the catechin equivalent to the CAE per 100 g of dry matter. The values of GAE or CAE within all samples are statistically significant *p* < 0.01 (ANOVA) with Tukey’s test, *n* > 3.

**Table 3:** Glucose, sucrose, and fructose content in coffee drinks.

Beverage	Concentration (mg/100 mL)		
	Sucrose	Glucose	Fructose
Coffee cherry shell	4.03 ± 0.17	170.81 ± 13.05	247.40 ± 46.11
Ethiopian Arabica beans	17.82 ± 8.32	2.93 ± 0.74	4.84 ± 0.17
Cascara-infused caffeine drink	17.09 ± 2.48	97.24 ± 7.79	166.06 ± 8.40

The values concentrations (sucrose, glucose or fructose) within all samples are statistically significant *p* < 0.01 (ANOVA) with Tukey’s test, *n* > 3.

## Data Availability

Additional data are available in the Supplementary Materials.

## Supplementary Materials

The following supporting information can be downloaded at: https://www.mdpi.com/article/10.3390/molecules30132749/s1, Figure S1: Chromatogram of the mixture of analytical standards at a concentration of 50 µg/mL; Figure S2: Chromatogram of a coffee infusion sample.
