# Supplementary material for: A Cascara-Infused Caffeine Drink as a Social Beverage
Source: Molecules. 2025 Jun 26;30(13):2749. doi: 10.3390/molecules30132749 (PMC12250676; doi:10.3390/molecules30132749)
Supplement: Supplementary file 1 [file molecules-30-02749-s001.zip › molecules-3678991-supplementary.pdf]

Supplementary file S1

Fig. S1. Chromatogram of the mixture of analytical standards at a concentration of 50 µg/ml: chlorogenic acid (Rt=4.667 min.), caffeic acid (Rt=5.350 min.), caffeine (Rt=6.113 min.)

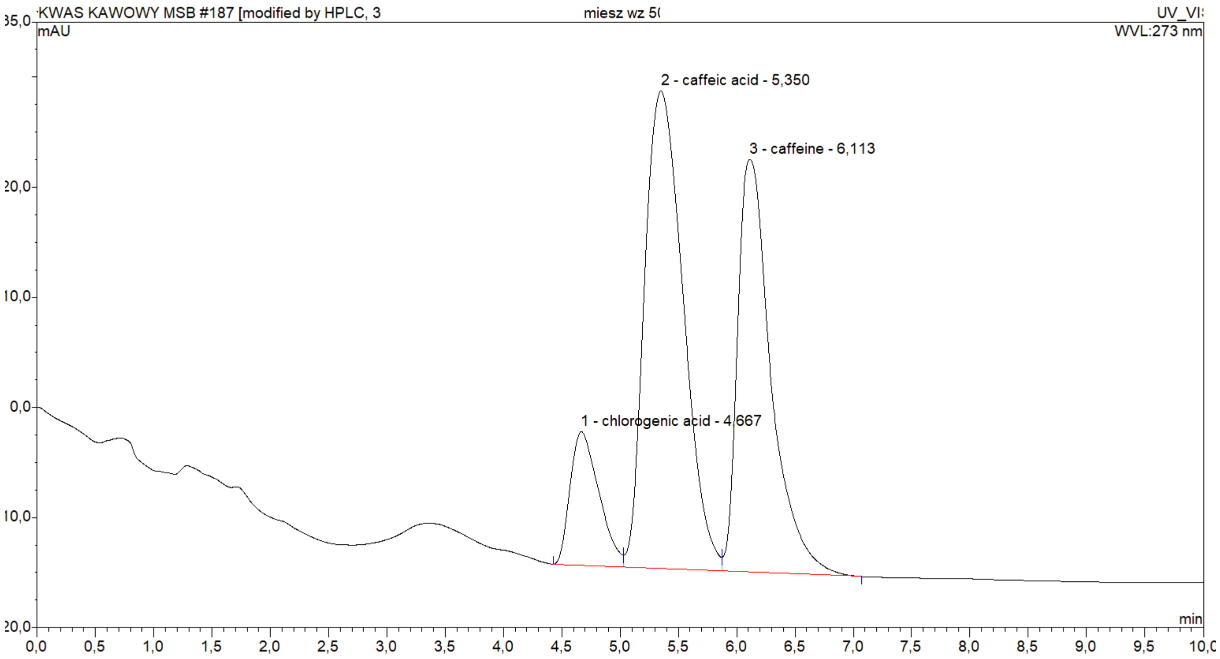

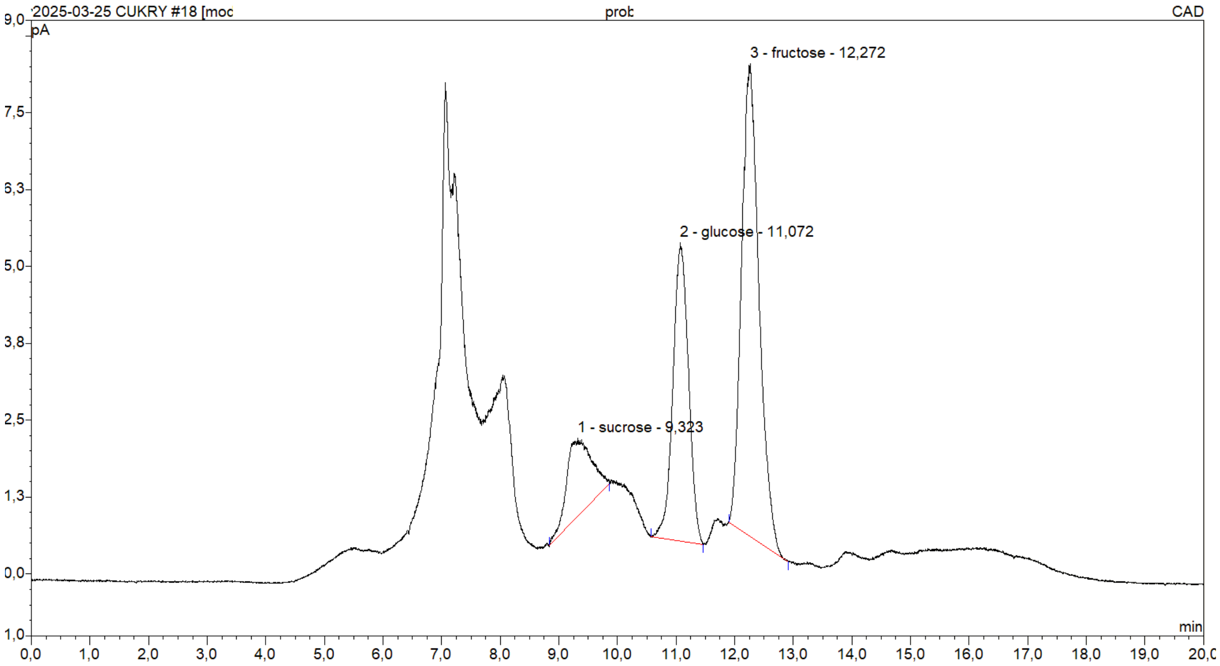

Fig. S2 Chromatogram of a coffee infusion sample: sucrose (Rt = 9.323 min.), glucose (Rt = 11.072min.), fructose (Rt = 12.272 min.).
